# Latent class analysis of depression among maintenance hemodialysis patients in China: a multicenter cross-sectional study

**DOI:** 10.1080/0886022X.2025.2556298

**Published:** 2025-09-09

**Authors:** Xiaoyu Chen, Peipei Han, Zhenwen Liang, Chen Yu, Kun Zhang, Siyi Zhu, Weijia Li, Yifan Xue, Qi Guo

**Affiliations:** ^a^ Department of Rehabilitation Medicine, Shanghai University of Medicine and Health Sciences Affiliated Zhoupu Hospital, Shanghai, China; ^b^ Department of Nephrology, Tongji Hospital, School of Medicine, Tongji University, Shanghai, China

**Keywords:** Depression, hemodialysis patients, latent class analysis, risk factors

## Abstract

**Background:**

Depression is a common mental disorder in hemodialysis patients. The present study aimed to identify subgroups of patients receiving hemodialysis based on depression and explore the influencing factors in a multicenter hemodialysis population in China.

**Methods:**

A total of 1,090 hemodialysis patients (682 men, mean aged 61.5 ± 12.6 years) from 7 facilities in Shanghai of China during 2020–2023. Depression was assessed by Patient Health Questionnaire 9 (PHQ-9). Latent class analysis (LCA) was performed to identify homogeneous groups of depressive symptoms. Analysis of variance and chi-square test were performed to establish class-dependent differences in depression severity. Multinomial logistic regression revealed the associations and related factors on most probable class.

**Results:**

Three latent classes were identified: High depressive symptoms (Class 1, *N* = 130, 11.9%), Moderate depressive symptoms (Class 2, *N* = 424, 38.9%) and Low depressive symptoms (Class 3, *N* = 536, 49.2%). The multinomial logistic regression results indicated that compared with the Class 3, the factors influencing depression in the Class 1 and Class 2 were age, body mass index (BMI), fall history, malnutrition and Charlson Comorbidity Index (CCI) in patients undergoing hemodialysis.

**Conclusions:**

The current study provides evidence for the heterogeneity of depression severity. A better understanding of depression risk factor profiles could help develop targeted prevention and intervention programs for the hemodialysis population.

## 1. Introduction

Depression is often the most common psychological complication in hemodialysis patients because of its high morbidity and adverse consequences [1]. Recent review reported that the overall prevalence of depression in patients receiving maintenance hemodialysis is 20–47% [2]. Depression increases the probability of malnutrition, inflammation, cardiovascular disease, and affects the prognosis of patients on hemodialysis. More seriously, the presence of depressive symptoms increased the risk of death in hemodialysis patients by 51% and was a significant factor in mortality [3]. Thus, it is necessary to investigate which individuals were at high risk of developing depressive symptoms among hemodialysis patients.

In the study of depression in hemodialysis patients, latent depressive states can be obtained indirectly by observing and measuring behavior. Although structured clinical interviews are the best way to determine depression status, most studies are based on medical records or a self-rating scale [3–5]. However, because the categorization criteria are too simple, it is impossible to distinguish the heterogeneity of depression. Surprisingly, latent classification analysis (LCA) can perfectly solve this problem and is a tool that can identify heterogeneity [6]. This method is superior to traditional methods, and can provide a more scientific method for the classification of the depression of hemodialysis patients.

A previous systematic review has already examined depressed risk in different subgroups in community-based samples and clinical depression populations. This review reported that the number of classes determined by the LCA models ranged from two to seven, especially models with three classes were the most common [7]. In addition, several recent studies have also applied LCA techniques to explore the heterogeneity of depression in different populations, such as children and adolescents [8], pregnant women [9], young men [10] and older adults [11]. These studies suggest that risk profiles in different risk domains provide meaningful information on the psychological health of different subgroups. Up to now, there have been no studies on the heterogeneity of depression using LCA techniques in patients undergoing maintenance hemodialysis.

To our knowledge, this study aimed to investigate profiles of risk factors for depression in the hemodialysis population. A better understanding of vulnerable groups could help develop targeted prevention or intervention programs that address the needs of these individuals. There is no latent category research on depression among hemodialysis groups. Therefore, this study aimed to use LCA to identify the factors influencing the depression in a multicenter hemodialysis population in China.

## 2. Methods

### 2.1. Participants

The Shanghai Hemodialysis Cohort Study (SHCS) is a multicenter prospective dynamic cohort study focusing on the relationships between physical performance and health status of a population receiving maintenance hemodialysis living in Shanghai, China (clinical trials. gov UMIN000056404). Our research population included maintenance hemodialysis patients from seven hospital in Shanghai of China who participated in and completed the questionnaire survey during 2020 and 2023. Inclusion criteria were (1) at least 18 years of age, (2) receiving maintenance hemodialysis for at least 3 months, (3) willingness to participate in the study. Participants with missing values in the dependent variable are excluded in the final analysis. The final analytic sample consisted 1090 patients (682 males and 408 females) after excluding 46 subjects. 38 participants refused to finish the depression questionnaire, 6 participants had missing data on falling evaluation, and 2 refused to participant in physical activity assessment or nutritional evaluation. All participants provided informed consent. The study was approved by the Ethics Committee of Shanghai University of Medicine and Health Sciences and the methods were carried out in accordance with the principles of the Declaration of Helsinki. The informed consent was obtained from all patients prior to enrollment in the study.

### 2.2. Depression

The patient Health Questionnaire-9 (PHQ-9) was used to assess depressive symptoms [12]. In research and clinical context, this questionnaire is often used as a brief diagnostic and severity measurement. To limit patient fatigue, all questionnaire testing, including the PHQ-9, was completed during the first hour of hemodialysis. This is also consistent with previous literature [13] and the details of measurement methods could be found in our previous study [4]. It was proved to be reliable and valid to detect depression determined by the PHQ-9 in dialysis patients (sensitivity of 92% and specificity of 92%, respectively) [14]. A recent systematic review involving 16 studies also recommended the PHQ-9 as free tool to assess depression in dialysis patients [15].

### 2.3. Risk factors

All patients were invited to receive a face-to-face interview and complete a standardized questionnaire. Risk factors included sociodemographic characteristics, health behaviors, blood markers and chronic disease condition. All categorical risk variables were summarized in Supplementary Table S1. Demographic characteristics consisted of age, sex, body mass index (BMI), education level, economic status, marital. status, children and living situation. BMI was computed from dry weight in kilograms divided by height in meters squared (kg/m^2^). The participants were categorized into four groups based on BMI as follows: underweight, BMI < 18.5 kg/m^2^; normal weight, 18.5≤ BMI < 23.9 kg/m^2^; overweight, 24.0 ≤BMI <27.9 kg/m^2^; and obese, BMI ≥ 28.0 kg/m^2^. Educational level is divided into the following three categories: less than high school, high school, and higher school. Economic status is categorized monthly household income into groups: <5,000 RMB/mo, 5,000–8,000 RMB/mo, 8,000–15,000 RMB/mo, and >15,000 RMB/mo. Marital status was classified as married (living together, divorced, separated, or widowed) or never married.

Health behaviors included smoking habits (current smoker or not), drinking habits (drinking alcohol once a week, drinking in the past, and never drinking were all considered as no drinking), fall history, nutritional status and physical activity. Malnutrition Inflammation Score (MIS) was used to evaluated the nutritional status, which is a comprehensive scoring system with significant associations with prospective hospitalization and mortality in patients undergoing hemodialysis [4]. Malnutrition was defined as MIS ≥ 6 in hemodialysis patients. A fall was defined as any event that results in a bodily change that forces an individual to unintentionally land on the ground or a lower level; the fall not to be caused by loss of consciousness, sudden onset paralysis, a violent blow, or epileptic seizure [16]. International Physical Activity Questionnaire (IPAQ) was used to assess physical activity [17].

Blood markers included hemoglobin, albumin, parathyroid hormone (PTH), calcium, phosphorus. All the blood sample were drawn prior to a dialysis. Fractional clearance index for urea (Kt/V) was used to evaluate the dialysis quality, which was monitored in every dialysis treatment using the OCM^®^ (On-line Clearance Monitor; Fresenius Medical Care, Bad Homburg, Germany).

Charlson Comorbidity Index (CCI) was conducted to evaluate the chronic disease condition [18]. CCI forms a composite score based on the presence of 19 comorbidities and is used to describe multiple comorbidities. We recoded this variable to the risk factor higher comorbidities (0–4 vs. > 4).

### 2.4. Statistical analysis

#### 2.4.1. Step 1: preparing the dataset

Data were aggregated and analyzed using SPSS (version 28.0) and Mplus (version 8.2). we only included cases with non-missing values (*N* = 1090). First, the nine components of PHQ-9 were classified as present or absent (1 or 0 as the categorical indicator variable) and analyzed using the LCA model. LCA is a common and useful human-centered approach that identifies individuals with similar characteristics and divides individuals into subclasses accordingly.

#### 2.4.2. Step 2: conducting the LCA

We applied LCA to identify whether there exist different clinical phenotypes of depressive symptoms in patients receiving maintenance hemodialysis. In our study, we fitted one to five latent class models to determine the optimal number of latent classes. Model fit indices used for the LCA included Log-likelihood (LL), Akaike information criterion (AIC), Bayesian information criterion (BIC), adjusted Bayesian Information Criterion (aBIC), Lo-Mendell-Rubin adjusted likelihood ratio (LMR), bootstrap likelihood ratio test (BLRT) and entropy [19]. For these fit indices, the model with the highest entropy and the lowest AIC and BIC is the optimal model. The entropy is an indicator of classification accuracy, with values close to 1 indicating greater accuracy. In this study, a three‐class model was determined as optimal based on fit indices.

Second, after the appropriate number of latent classes has been identified, hemodialysis patients were assigned to their most likely subgroup based on their highest posterior class probability. Analysis of Variance (ANOVA) and Chi-square tests were used to examine the distribution of related risk factors. Subsequently, multinomial logistic regression was performed to estimate the correlates of related risk factors with subtypes.

#### 2.4.3. Step 3: conducting a sensitivity analysis

We only included cases with non-missing values (*N* = 1,090) in the dependent variables in our study. To ensure that the exclusion of *n* = 46 cases did not lead to substantial changes in the LCA results (e.g. class structure), we ran the LCA with all cases (*N* = 1,136) again. Then, we compared it to the results of the LCA model, where we only included cases with non-missing values in the dependent variables (*N* = 1,090).

## 3. Results

### 3.1. LCA results

The model estimates 1 to 5 classes. Model fit indices for various models with different latent classes are listed in Table 1. The results showed that the AIC, BIC, and aBIC decreased with the increase of classification number from 1 class to 3 classes. In the 4-class and 5-class models, the LMR and BLRT values were not significant. Therefore, the best models were selected for the three categories (Table 1).

**Table 1.** Fitness indicators of different latent class models (*N* = 1090).

	LL	AIC	BIC	aBIC	Entropy	LMR	BLRT	Class size and assignment probability
1 class	−5704.058	11426.116	11471.061	11442.475	–	–	–	1090 (100%)
2 class	−4882.774	9803.549	9898.433	9838.085	0.819	<0.001	<0.001	389 (35.69%)/701 (64.31%)
**3 class**	**−4762.389**	**9582.777**	**9727.601**	**9635.491**	**0.775**	**<0.001**	**<0.001**	**130(11.93%)/424(38.90%)/536(49.17%)**
4 class	−4733.163	9544.326	9739.089	9615.217	0.760	0.193	0.197	147(13.49%)/366(33.58%)/51(4.68%)/526(48.26%)
5 class	−4706.829	9511.658	9756.361	9600.726	0.706	0.371	0.376	94(8.62%)/186(17.06%)/53(4.86%)/330(30.28%)/427(39.17%)

Notes. LL, Log-likelihood; AIC, Akaike Information Criterion; BIC, Bayesian Information Criterion; aBIC, Adjusted Bayesian Information Criterion; LMR, Lo-Mendell-Rubin adjusted likelihood ratio; BLRT, bootstrap likelihood ratio test.

The sensitivity analysis (Supplement Table S2) did not reveal any differences in the results when only including cases with non-missing values in the dependent variables (*N* = 1136) vs. all cases (*N* = 1090). Thus, we assumed that only using cases with non-missing values in the dependent variables would not bias our LCA results.

**Table 2.** Characteristics of respondents across latent classes.

Characteristics	Total	High depressive symptoms (Class 1)	Moderate depressive symptoms (Class 2)	Low depressive symptoms (Class 3)	*P*
	(*N* = 1,090)	(*N* = 130)	(*N* = 424)	(*N* = 536)	
Age (y)	61.5 ± 12.6	63.1 ± 13.3	60.5 ± 13.0	61.8 ± 12.0	0.090
Age classification (%)					0.009
20–44 y	124 (11.4)	17 (13.1)	57 (13.4)	50 (9.3)	
45–59 y	291 (26.7)	20 (15.4)	116 (27.4)	155 (28.9)	
≥60 y	675 (61.9)	93 (71.5)	251 (59.2)	331 (61.8)	
Sex (%)					0.232
Male	682 (62.6)	87 (66.9)	253 (59.7)	342 (63.8)	
Female	408 (37.4)	43 (33.1)	171 (40.3)	194 (36.2)	
BMI (%)					0.015
Underweight (< 18.5 kg/m^2^)	93 (8.5)	9 (6.9)	46 (10.8)	38 (7.1)	
Normal weight (18.5–23.9 kg/m^2^)	579 (53.1)	74 (56.9)	241 (56.8)	264 (49.3)	
Overweight (24.0–27.9 kg/m^2^)	120 (11.0)	15 (11.5)	40 (9.4)	65 (12.1)	
Obese (≥28.0 kg/m^2^)	298 (27.3)	32 (24.6)	97 (22.9)	169 (31.5)	
Education (%)					0.038
Less than high school	254 (23.3)	42 (32.3)	94 (22.2)	118 (22.0)	
High school	661 (60.6)	74 (56.9)	251 (59.2)	336 (62.7)	
Higher school	175 (16.1)	14 (10.8)	79 (18.6)	82 (15.3)	
Economic status (%)					0.137
<5,000 RMB/mo	280 (25.7)	38 (29.2)	112 (26.4)	130 (24.3)	
5,000–8,000 RMB/mo	303 (27.8)	44 (33.8)	110 (25.9)	149 (27.8)	
8,000–15,000 RMB/mo	319 (29.3)	34 (26.2)	133 (31.4)	152 (28.4)	
>15,000 RMB/mo	188 (17.2)	14 (10.8)	69 (16.3)	105 (19.6)	
Widowed (%)	85 (7.8)	8 (6.2)	37(8.7)	40 (7.5)	0.582
Being childless(%)	76 (7.0)	12 (9.2)	32 (7.5)	32 (6.0)	0.356
Living situation(%)	58 (5.3)	4 (3.1)	28 (6.6)	26 (4.9)	0.232
Drinking (%)					0.525
Never or former	656 (60.2)	119 (91.5)	381 (89.9)	483 (90.1)	
<7 d/wk	95 (8.7)	9 (6.9)	37 (8.7)	49 (9.1)	
Daily	12 (1.1)	2 (1.5)	6 (1.4)	4 (0.7)	
Smoking (%)					0.705
Never	564 (51.7)	66 (50.8)	229 (54.0)	269 (50.2)	
Former	285 (26.1)	35 (26.9)	101 (23.8)	149 (27.8)	
Current	241 (22.1)	29 (22.3)	94 (22.2)	118 (22.0)	
Fall History (%)	301 (27.6)	54 (41.5)	121 (28.5)	126 (23.5)	<0.001
Malnutrition (%)	307 (28.2)	47 (36.2)	137 (32.3)	123 (22.9)	<0.001
IPAQ (Met-min/wk)	1302 (462,2772)	1015 (334,2519)	1133 (258,2772)	1386 (495,3159)	0.184
CCI					0.038
0–4	694 (63.7)	70 (53.8)	271 (63.9)	353 (65.9)	
>4	396 (36.3)	60 (46.2)	153 (36.1)	183 (34.1)	
Laboratory parameters					
Hemoglobin (g/dL)	110.50 ± 16.73	111.8 ± 17.7	110.0 ± 16.4	110.6 ± 16.7	0.564
Albumin (g/L)	39.28 ± 3.75	39.40 ± 3.88	39.34 ± 3.87	39.20 ± 3.75	0.786
PTH (pg/dL)	345.59 ± 314.71	375.22 ± 324.85	360.90 ± 315.24	326.31 ± 311.19	0.130
Calcium (mg/dL)	2.26 ± 0.25	2.27 ± 0.26	2.26 ± 0.22	2.25 ± 0.25	0.527
Phosphorus (mg/dL)	1.98 ± 0.65	1.99 ± 0.68	1.95 ± 0.66	2.00 ± 0.63	0.518
Kt/v	1.36 ± 0.34	1.31 ± 0.29	1.38 ± 0.37	1.36 ± 0.34	0.090
PHQ-9	4.78 ± 5.05	13.85 ± 5.40	6.51 ± 3.12	1.21 ± 1.46	<0.001

Notes. BMI, body mass index; IPAQ, international physical activity questionnaire; Met-min/wk, metabolic equivalent task minutes per week; CCI, Charlson Comorbidity Index; PTH, parathyroid hormone; Kt/V, fractional clearance index for urea; PHQ-9, Patient Health Questionnaire-9.

### 3.2. Definition of latent class

**Figure 1.**
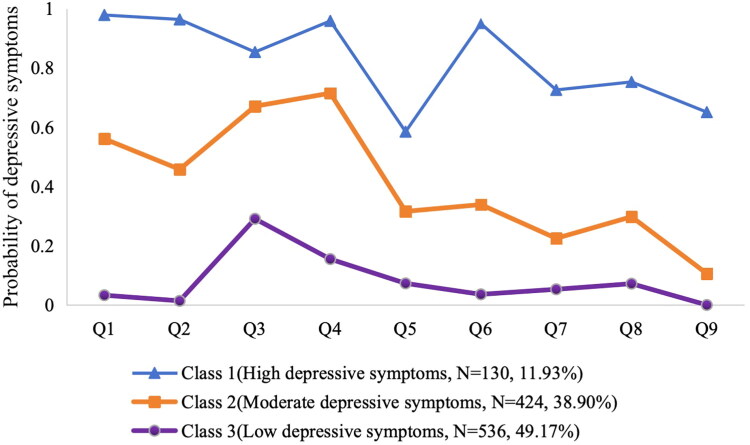
Response patterns for latent classes.

As shown in Table 1, the three potential classes are included: Class 1(*N* = 130, 11.9%), Class 2 (*N* = 424, 38.9%), Class 3 (*N* = 536,49.2%). In Figure 1. we present the means of dichotomized PHQ-9 items using the score (0-1-1-1) approach. The probability of latent class response Yes is shown in Figure 1: (1) High depressive symptoms class (Class 1): The score probability in this class was high, which suggested that the hemodialysis patients in this category had high depressive symptoms, so they were labeled the “High depressive symptoms class”. (2) Moderate depressive symptoms class (Class 2): The second class had a moderate score probability of depression, which was lower than that observed in the first class and higher than that observed in the third class. So, Class 2 was named the “Moderate depressive symptoms class”. (3) Low depressive symptoms class (Class 3), which was characterized by members of this group having the lowest scores on the depressive symptoms among the three groups, so the Class 3 was named the “Low depressive symptoms class”.

### 3.3. Univariate analysis of latent class of depression

As shown in Table 2, BMI, education, fall history, malnutrition and CCI were significantly different across the three groups (*p* < 0.05). A higher proportion of patients in the first category had education below high school level. In addition, Class 1 had the highest percentage of fall history and malnutrition than other classes. Class 1 reported the highest mean CCI. The results of ANOVA showed that the PHQ-9 were significantly different (*p* < 0.001). In other words, depression was significantly different in classes, and it can be seen from the post-test results that Class 1 scored significantly higher than Class 2 and Class 3 on the three variables. The sample of hemodialysis patients for the qualitative study is presented in Table 2.

### 3.4. Multinomial logistic regression analysis of latent classes of depression

Taking the low depressive symptoms class (Class 3) as the reference class, the high depressive symptoms class (Class 1), and the moderate depressive symptoms class (Class 2) were compared. Odds ratio (OR) results showed that the latent category of depression was influenced by factors such as age, BMI, fall history, malnutrition and CCI. Table 3 showed the results.

**Table 3.** Multinomial regression analysis of different classes with class 3 as reference. Results are shown as odds ratios with 95% confidence intervals.

	High depressive symptoms (Class 1)			Moderate depressive symptoms (Class 2)			VIF
	OR	CI (95%)	*P*	OR	CI (95%)	*P*	
Age							1.186
20–44 y	2.03	1.04–3.95*	0.038	1.61	1.02–2.54*	0.040	
45–59 y	0.62	0.36–1.06	0.080	1.06	0.78–1.44	0.706	
≥60 y	1			1			
BMI							1.027
Underweight (< 18.5 kg/m^2^)	0.68	0.30–1.50	0.335	1.12	0.69–1.80	0.655	
Normal weight (18.5–23.9 kg/m^2^)	1			1			
Overweight (24.0–27.9 kg/m^2^)	0.75	0.40–1.43	0.381	0.64	0.41–0.99*	0.044	
Obese (≥28.0 kg/m^2^)	0.66	0.41–1.05	0.077	0.64	0.47–0.88*	0.005	
Education							1.108
Less than high school	2.00	0.97–4.11	0.059	0.87	0.56–1.36	0.547	
High school	1.32	0.69–2.53	0.401	0.81	0.56–1.17	0.260	
Higher school	1			1			
Fall History							1.031
Yes	2.08	1.37–3.14***	<0.001	1.27	0.94–1.72	0.115	
No	1			1			
Malnutrition							1.041
Yes	1.61	1.04–2.47*	0.031	1.50	1.11–2.02*	0.008	
No	1			1			
CCI							1.074
0–4	1			1			
>4	1.56	1.03–2.36*	0.034	1.16	0.87–1.53	0.313	

Notes. *p < 0.05. ***p < 0.001.

OR, odds ratio; CI, confidence interval; BMI, body mass index; VIF, variance inflation factor; CCI, Charlson Comorbidity Index.

Compared with participants aged over 60 years, patients aged 20 to 44 years were 2 times more likely to belong to Class 1 than Class 3 (OR: 2.03, *p* = 0.038, CI: 1.04–3.95), and 1.6 times more likely to belong to Class 2 than Class 3 (OR: 1.61, *p* = 0.040, CI: 1.02–2.53).

Compared with normal weight, the participants with overweight and obesity were similarly less likely to belong to Class 2 than Class 3 (overweight: OR: 0.64, *p* = 0.044, CI: 0.41–0.99; obese: OR: 0.64, *p* = 0.005, CI: 0.47–0.88), while BMI classifications were not significantly different between C1 and C3. So, overweigh and obesity can only predict Class 2 but not any other class.

Patients who had fall history were 2 times more likely to belong to class members that were Class 1 (OR: 2.08, *p* < 0.001, CI: 1.37–3.14), rather than Class 2. Compared with less comorbidities, the probability of more comorbidities belonging to Class 1 was higher (OR: 1.56, *p* = 0.034; CI:1.03–2.36). And Class 2 and Class 3 were not significantly different by CCI. So, fall history and CCI can only predict Class 1 but not any other class members.

Compared with normal nutritional status, the participants with malnutrition were inclined to belong to Class 1 which was 1.6 times that of Class 3 (OR:1.61, *p* = 0.031; CI:1.04–2.47), and the tendency to belong to Class 2 which was 1.5 times that of Class 3 (OR: 1.50, *p* = 0.008; CI:1.11–2.02).

### 3.5. Relationships between profiles of risk factors, depressive, and anxiety symptoms

The mean scores of depressive symptoms for the three identified risk profiles are shown in Table 2. Individuals in the high depressive symptoms class descriptively reported the highest levels of depressive symptoms. Individuals in the moderate depressive symptoms class had the second highest symptom levels, while those in the low depressive symptoms had the lowest scores. Multiple group analyses revealed statistically significant differences in depressive symptoms (χ^2^ = 432.13, *p* < 0.001) among the three profiles (Supplement Table S3).

## 4. Discussion

To the best of our knowledge, this study aimed to examine the heterogeneity of depression among hemodialysis patients and delineate the characteristics of subtypes at different depressive symptoms classes, using LCA in a Chinese multicenter hemodialysis population. LCA is an important psychological research method that assumes that individuals can be categorized into groups with similar patterns of several behaviors based on how they respond to a set of observed indicators [6]. Our results suggest that depression in hemodialysis patients is heterogeneous, and has potential characteristics. There are three subtypes in the population, namely, high depressive symptoms class, moderate depressive symptoms class, and low depressive symptoms class. This is consistent with previous studies involving LCA, which divided mental health into three classes [20,21].

Most hemodialysis patients in this study belong to the “low depressive symptoms class”, and the prevalence of “moderate depressive symptoms class” and “high depressive symptoms class” were 11.9% and 38.9%, respectively. Overall, the prevalence of depression is around 50%. This prevalence was consistent with previous studies. Nguyen et al. [22] and Dang et al. [23] reported that the prevalence of depression in hemodialysis patients were 42.5% and 41.8%, respectively. However, further longitudinal studies are needed to determine changes in prevalence.

Furthermore, age, BMI, fall history, malnutrition and CCI are predictors of the latent class of depression. This phenomenon elucidated that the better understanding of predictors about depression for hemodialysis patients, the more active medical staffs are in coping with the mental disorders. Therefore, enhancing the cognition of mental health of medical staff and patients is beneficial to the improvement of depressive state. The relevant departments of hospitals should screen the related risk factors of depression in advance, so as to screen out patients at high risk of depression, and actively implement targeted psychological interventions and health education measures.

Examining differences between the classes in terms of age suggested that aged 20–44 years is a stronger predictor for depression in both Class 1 and Class 2 than those over 60 years old. These results might suggest that depression is more frequent in younger patients compared with elderly patients. This is consistent with previous findings suggesting that there is an association of younger age with poor psychological disorder, such as depression [24,25]. This may be due to the fact that aging is often considered as inevitably linked to health problems. For both adolescents and adults, reconciliation with health issues is more difficult, and patients often perceive it as a disability and impairment. Therefore, younger patients need more ESRD-oriented support to alleviate their health-related complaints and require extensive psychological assistance to cope with the negative emotions associated with the disease than older adults.

A large number of previous studies have shown that in the various population, obesity can be a risk factor for depression [26] and can also act as a protective factor for depression [27,28], which is known as the obesity paradox. Notably, there are two previous studies that have explored the association among dialysis population, and demonstrated that obesity is a risk factor for depression [29,30]. Although there are still no studies enrolling obesity as a protective factor for depression in hemodialysis patients, there are relevant findings. Beberashvili et al. [31] suggested that maintenance hemodialysis patients with sarcopenic obesity have better nutritional status and lower all-cause mortality versus nonobese sarcopenia. In addition, Villain et al. [32] reported that there is a survival benefit from obesity measured by BMI in elderly hemodialysis patients. Similarly, the obesity paradox has been proposed in end-stage renal disease patients [33]. Consistent with our study, hemodialysis patients are more likely to have poor outcomes when BMI is low, and obesity has a favorable impact on their future health. Protein-energy wasting (PEW) and inflammation might be explanations. Patients who have lower BMI may have PEW that is responsible for increased poor outcomes. Moreover, the inflammatory response may be an important factor. Our analysis of C-reactive protein (CRP) revealed higher levels in malnourished individuals compared to non-malnourished individuals[medians ±25–75th percentiles for CRP, 3.15(1.27,9.78)vs 2.29(1.18,5.43), *p* < 0.001]. Therefore, we believe that malnourished individuals are more susceptible to the disruption by inflammatory processes [34]. Therefore, obesity may potentially attenuate the degree of PEW and/or inflammation, which can be beneficial to hemodialysis patients. Our results contribute to some extent to the study of the association between obesity and mental health in the hemodialysis population.

In the current study, fall history has been reported to have twice times risk for depression belonging to Class 1 when compared to Class 3. This is in line with previous findings. A study conducted in Korea found a strong relationship between experiences of falling and depression [35], and a recent Chinese study involving 11857 older adults demonstrated a link between falls and depression [36]. Interestingly, however, the results from a meta-analysis unveil the lack of relationship between falls and depression [37]. In fact, the most likely bidirectional relationship between falls and depression therefore appears to be very complex. Although the exact mechanisms underlying the association between fall and depressive symptoms is not fully understood, there are many possible explanations for the relationship. Epidemiologic evidence reported that depressive symptoms in older adults are associated with a large number of known fall risk factors, including psychomotor retardation, which can lead to decreased walking speed and poor balance. Another possible explanation could be that depression is often frequently accompanied by a reduction in appetite with a consequent loss of weight and muscle mass, which may also increase the risk of falling. Notably, this study explored the relationship between falls and depression in hemodialysis patients. Thus, based on the results of this study, Chinese government may require to establish a systematic social support system to prevent fall accidents especially in hemodialysis patients. In addition, this cross-sectional study could not hypothesize a causal association, and longitudinal studies are needed to further investigate the relationship in the future.

Our study confirmed that malnourished patients in both the high and moderate depressive symptoms groups had about a 1.5-fold higher risk of depression compared to those in the low depressive symptoms class, which are consistent with previous findings [4,38,39]. Loss of appetite and reduced dietary adherence may partly explain the relationship between malnutrition and depression. However, there are also conflicting results [40,41]. The inconsistent findings may be due to the small sample size of individual dialysis centers and incomplete measurements to assess nutritional status in previous studies. The gut microbiome-brain axis is also one of the current research hotspots. During the subsequent follow-up period, we collected fecal samples from some patients. In the future, we will further examine the gut microbiota to explore the relationship between nutrition status and depression.

The results demonstrated that patients undergoing hemodialysis presented more comorbidities, as reflected by higher scores of CCI, had higher risk of depression. The presence of multiple comorbidities increases the burden of illness, such as functional impairment and selfcare demands, resulting in depression and poor therapeutic adherence [42,43]. Our findings are further strengthened by recent studies [44,45], which showed that higher scores of CCI were related to more prevalence of depression among patients receiving maintenance hemodialysis. Taken together, these results suggest that the depression of hemodialysis patients may be highly heterogeneous and likely to be affected by the co-present of chronic somatic diseases. However, whether the association between the CCI and depression is mediated by the burden of treatment (e.g. medication complexity) or physiological stress (e.g. oxidative stress) still requires further research to explore in the future.

Our study has several strengths, including the application of LCA technology in a large sample size of hemodialysis patients and consideration of risk patterns rather than single risk factors. Nevertheless, the potential limitations should be considered in the present study. First, this was a cross-sectional study and cannot infer a causal relationship. In the future, we will conduct in-depth longitudinal cohort studies to verify the stability of these latent classes. Secondly, LCA method has significance limitations, and the clinical utility of the resulting latent subtypes remains unclear. For example, LCA method is still evolving and from clinical perspectives, research findings based on LCA methods have not improved the treatment of depression. Moreover, available LCA guidelines are lacking adequate information on how to validate latent subtypes. Thirdly, the mental health measurement tools used in this study were all self-rating scales, which may affect the accuracy of the results. In the future, we plan to incorporate objective parameters (e.g. voice analysis/motion sensor data), and we will even seek the cooperation of psychologists to further enhance the accuracy of depression diagnosis. Fourth, we had to handle the risk factors of LCA as categorical variables, which reduces the information richness of the data. Choosing different re-encodings may lead to different results. We included only a limited number of risk factors for depression in the LCA. There may be more risk factors that explain variations in the development of depressive symptoms, such as social and family support, and dietary patterns. In addition, this study relies on subjective reports of subjects. It ignores the data bias caused by personal shame, prejudice, and other factors, which cover up their depressive symptoms or lack of awareness of their feelings. Although the results of this study are statistically significant, only patients from seven hospital in Shanghai of China were selected for the study, and the results may not be extended to other populations (such as mental disorders, and people from other countries). Future studies could include more evidence-based risk factors in the LCA to analyze how much this will change the class structure.

## 5. Conclusion

In conclusion, this study using LCA to explore depression among maintenance hemodialysis patients, and the study revealed three subgroups differing in symptoms severity of depression (High depressive symptoms, Moderate depressive symptoms, Low depressive symptoms). Age, BMI, fall history, malnutrition and CCI are predictors of the latent class of depression. The findings highlight the necessity for early assessment to identify hemodialysis patients at high risk for depression, followed by interventions that are meticulously tailored to address the unique symptom presentations, which may be a potential strategy for preventing depression among Chinese hemodialysis patients.

## Supplementary Material

Supplementary_material_clean.docx

## Data Availability

The datasets used and/or analyzed during the current study are available from the corresponding author upon reasonable request.

## References

[1] King-Wing Ma T, Kam-Tao Li P. Depression in dialysis patients. Nephrology (Carlton). 2016;21(8):639–646. doi: 10.1111/nep.12742. 26860073

[2] Li Y, Zhu B, Shen J, et al. Depression in maintenance hemodialysis patients: what do we need to know? Heliyon. 2023;9(9):e19383. doi: 10.1016/j.heliyon.2023.e19383. 37662812 PMC10472011

[3] Farrokhi F, Abedi N, Beyene J, et al. Association between depression and mortality in patients receiving long-term dialysis: a systematic review and meta-analysis. Am J Kidney Dis. 2014;63(4):623–635. doi: 10.1053/j.ajkd.2013.08.024. 24183836

[4] Chen X, Han P, Song P, et al. Mediating effects of malnutrition on the relationship between depressive symptoms clusters and muscle function rather than muscle mass in older hemodialysis patients. J Nutr Health Aging. 2022;26(5):461–468. doi: 10.1007/s12603-022-1778-8.35587758

[5] Zhang S, Liu SX, Wu QJ, et al. Association between handgrip strength and depressive symptoms in patients undergoing hemodialysis: a cross-sectional study from a single Chinese center. BMC Psychiatry. 2024;24(1):182. doi: 10.1186/s12888-024-05576-8. 38443831 PMC10913615

[6] Wang J, Xu M, Li X, et al. A latent class analysis of hopelessness in relation to depression and trauma during the COVID-19 pandemic in China. J Affect Disord. 2023;329:81–87. doi: 10.1016/j.jad.2023.02.077. 36841301 PMC9951088

[7] Ulbricht CM, Chrysanthopoulou SA, Levin L, et al. The use of latent class analysis for identifying subtypes of depression: a systematic review. Psychiatry Res. 2018;266:228–246. doi: 10.1016/j.psychres.2018.03.003. 29605104 PMC6345275

[8] Göbel K, Cohrdes C. The whole is greater than the sum of its parts: profiles of multiple mental health risk factors using Latent class analysis. Child Adolesc Psychiatry Ment Health. 2021;15(1):27. doi: 10.1186/s13034-021-00380-8. 34127038 PMC8204434

[9] Sun JW, Cao DF, Li JH, et al. Profiles and characteristics of clinical subtypes of perinatal depressive symptoms: a latent class analysis. J Adv Nurs. 2019;75(11):2753–2765. doi: 10.1111/jan.14136. 31236991

[10] Wahid SS, Sandberg J, Sarker M, et al. A distress-continuum, disorder-threshold model of depression: a mixed-methods, latent class analysis study of slum-dwelling young men in Bangladesh. BMC Psychiatry. 2021;21(1):291. doi: 10.1186/s12888-021-03259-2. 34088289 PMC8178879

[11] Wang L-Q, Zhang T-H, Dang W, et al. Heterogenous subtypes of late-life depression and their cognitive patterns: a latent class analysis. Front Psychiatry. 2022;13:917111. doi: 10.3389/fpsyt.2022.917111. 35873245 PMC9298648

[12] Kroenke K, Spitzer RL, Williams JB. The PHQ-9: validity of a brief depression severity measure. J Gen Intern Med. 2001;16(9):606–613. doi: 10.1046/j.1525-1497.2001.016009606.x. 11556941 PMC1495268

[13] Agganis BT, Weiner DE, Giang LM, et al. Depression and cognitive function in maintenance hemodialysis patients. Am J Kidney Dis. 2010;56(4):704–712. doi: 10.1053/j.ajkd.2010.04.018. 20673602 PMC2943330

[14] Watnick S, Wang PL, Demadura T, et al. Validation of 2 depression screening tools in dialysis patients. Am J Kidney Dis. 2005;46(5):919–924. doi: 10.1053/j.ajkd.2005.08.006. 16253733

[15] Kondo K, Antick JR, Ayers CK, et al. Depression screening tools for patients with kidney failure: a systematic review. Clin J Am Soc Nephrol. 2020;15(12):1785–1795. doi: 10.2215/CJN.05540420. 33203736 PMC7769028

[16] Lamb SE, Jørstad-Stein EC, Hauer K, Prevention of Falls Network E, Outcomes Consensus G, et al. Development of a common outcome data set for fall injury prevention trials: the Prevention of Falls Network Europe consensus. J Am Geriatr Soc. 2005;53(9):1618–1622.,. doi: 10.1111/j.1532-5415.2005.53455.x. 16137297

[17] Bassett DR. Jr International physical activity questionnaire: 12-country reliability and validity. Med Sci Sports Exerc. 2003;35(8):1396. doi: 10.1249/01.MSS.0000078923.96621.1D. 12900695

[18] Liu H, Song B, Jin J, et al. Length of stay, hospital costs and mortality associated with comorbidity according to the Charlson comorbidity index in immobile patients after ischemic stroke in China: a national study. Int J Health Policy Manag. 2022;11(9):1780–1787. doi: 10.34172/ijhpm.2021.79. 34380205 PMC9808248

[19] Owczarek M, Jurek J, Nolan E, et al. Nutrient deficiency profiles and depression: a latent class analysis study of American population. J Affect Disord. 2022;317:339–346. doi: 10.1016/j.jad.2022.08.100. 36049605

[20] Liu Z, Liu R, Zhang Y, et al. Latent class analysis of depression and anxiety among medical students during COVID-19 epidemic. BMC Psychiatry. 2021;21(1):498. doi: 10.1186/s12888-021-03459-w. 34641795 PMC8506472

[21] Kenntemich L, von Hülsen L, Schäfer I, et al. Profiles of risk factors for depressive and anxiety symptoms during the COVID-19 pandemic: a latent class analysis. Psychiatry Res. 2023;323:115150. doi: 10.1016/j.psychres.2023.115150. 36913873 PMC9985930

[22] Nguyen TTN, Liang SY, Liu CY, et al. Self-care self-efficacy and depression associated with quality of life among patients undergoing hemodialysis in Vietnam. PLoS One. 2022;17(6):e0270100. doi: 10.1371/journal.pone.0270100. 35709232 PMC9202875

[23] Dang LT, Luong TC, Nguyen DH, et al. The associations of suspected COVID-19 symptoms with anxiety and depression as modified by hemodialysis dietary knowledge: a multi-dialysis center study. Nutrients. 2022;14(12):2364. doi: 10.3390/nu14122364. 35745093 PMC9230868

[24] Valderrábano F, Jofre R, López-Gómez JM. Quality of life in end-stage renal disease patients. Am J Kidney Dis. 2001;38(3):443–464. doi: 10.1053/ajkd.2001.26824. 11532675

[25] Laudański K, Nowak Z, Niemczyk S. Age-related differences in the quality of life in end-stage renal disease in patients enrolled in hemodialysis or continuous peritoneal dialysis. Med Sci Monit. 2013;19:378–385. doi: 10.12659/MSM.883916. 23685340 PMC3665666

[26] Rao WW, Zong QQ, Zhang JW, et al. Obesity increases the risk of depression in children and adolescents: results from a systematic review and meta-analysis. J Affect Disord. 2020;267:78–85. doi: 10.1016/j.jad.2020.01.154. 32063576

[27] Zhang L, Liu K, Li H, et al. Relationship between body mass index and depressive symptoms: the “fat and jolly” hypothesis for the middle-aged and elderly in China. BMC Public Health. 2016;16(1):1201. doi: 10.1186/s12889-016-3864-5. 27894296 PMC5126817

[28] Oh J, Chae JH, Kim TS. Age-specific association between body mass index and depression: the Korea National Health and Nutrition Examination Survey 2014. Int J Obes (Lond). 2018;42(3):327–333. doi: 10.1038/ijo.2017.234. 28974741

[29] Vučković M, Radić J, Kolak E, et al. Body composition parameters correlate to depression symptom levels in patients treated with hemodialysis and peritoneal dialysis. Int J Environ Res Public Health. 2023;20(3):2285. doi: 10.3390/ijerph20032285. 36767652 PMC9915081

[30] Liu WJ, Musa R, Chew TF, et al. DASS21: A useful tool in the psychological profile evaluation of dialysis patients. Am J Med Sci. 2018;355(4):322–330. doi: 10.1016/j.amjms.2017.11.015. 29661345

[31] Beberashvili I, Azar A, Khatib A, et al. Sarcopenic obesity versus nonobese sarcopenia in hemodialysis patients: differences in nutritional status, quality of life, and clinical outcomes. J Ren Nutr. 2023;33(1):147–156. doi: 10.1053/j.jrn.2022.05.003. 35597322

[32] Villain C, Ecochard R, Genet L, et al. Impact of BMI variations on survival in elderly hemodialysis patients. J Ren Nutr. 2015;25(6):488–493. doi: 10.1053/j.jrn.2015.05.004. 26139338

[33] Park J, Ahmadi SF, Streja E, et al. Obesity paradox in end-stage kidney disease patients. Prog Cardiovasc Dis. 2014;56(4):415–425. doi: 10.1016/j.pcad.2013.10.005. 24438733 PMC4733536

[34] Kalantar-Zadeh K, Kopple JD. Relative contributions of nutrition and inflammation to clinical outcome in dialysis patients. Am J Kidney Dis. 2001;38(6):1343–1350. doi: 10.1053/ajkd.2001.29250. 11728973

[35] Kim JH. Experiences of falling and depression: results from the Korean Longitudinal Study of Ageing. J Affect Disord. 2021;281:174–182. doi: 10.1016/j.jad.2020.12.026. 33321383

[36] Wang J, Li S, Hu Y, et al. The moderating role of psychological resilience in the relationship between falls, anxiety and depressive symptoms. J Affect Disord. 2023;341:211–218. doi: 10.1016/j.jad.2023.08.060. 37579882

[37] Gambaro E, Gramaglia C, Azzolina D, et al. The complex associations between late life depression, fear of falling and risk of falls. A systematic review and meta-analysis. Ageing Res Rev. 2022;73:101532. doi: 10.1016/j.arr.2021.101532. 34844015

[38] Markaki AG, Charonitaki A, Psylinakis E, et al. Nutritional status in hemodialysis patients is inversely related to depression and introversion. Psychol Health Med. 2019;24(10):1213–1219. doi: 10.1080/13548506.2019.1612074. 31046446

[39] Koo J-R, Yoon J-W, Kim S-G, et al. Association of depression with malnutrition in chronic hemodialysis patients. Am J Kidney Dis. 2003;41(5):1037–1042. doi: 10.1016/s0272-6386(03)00201-4. 12722038

[40] Oliveira CM, Costa SP, Costa LC, et al. Depression in dialysis patients and its association with nutritional markers and quality of life. J Nephrol. 2012;25(6):954–961. doi: 10.5301/jn.5000075. 22241638

[41] Barros A, da Costa BE, Poli-de-Figueiredo CE, et al. Nutritional status evaluated by multi-frequency bioimpedance is not associated with quality of life or depressive symptoms in hemodialysis patients. Ther Apher Dial. 2011;15(1):58–65. doi: 10.1111/j.1744-9987.2010.00874.x. 21272254

[42] Shirazian S. Depression in CKD: understanding the mechanisms of disease. Kidney Int Rep. 2019;4(2):189–190. doi: 10.1016/j.ekir.2018.11.013. 30775614 PMC6365402

[43] Hammer-Helmich L, Haro JM, Jonsson B, et al. Functional impairment in patients with major depressive disorder: the 2-year PERFORM study. Neuropsychiatr Dis Treat. 2018;14:239–249. doi: 10.2147/NDT.S146098. 29386897 PMC5767094

[44] Jeon YH, Lim J-H, Jeon Y, et al. The impact of severe depression on the survival of older patients with end-stage kidney disease. Kidney Res Clin Pract. 2024;43(6):818–828. doi: 10.23876/j.krcp.22.268. 37644771 PMC11615450

[45] Ye W, Wang L, Wang Y, et al. Depression and anxiety symptoms among patients receiving maintenance hemodialysis: a single center cross-sectional study. BMC Nephrol. 2022;23(1):417. doi: 10.1186/s12882-022-03051-8. 36585621 PMC9804950

