# Supplementary material for: Latent class analysis of depression among maintenance hemodialysis patients in China: a multicenter cross-sectional study
Source: Ren Fail. 2025 Sep 9;47(1):2556298. doi: 10.1080/0886022X.2025.2556298 (PMC12422040; doi:10.1080/0886022X.2025.2556298)
Supplement: Supplementary_material_clean.docx [file IRNF_A_2556298_SM3098.docx]

**Appendix. Supplementary materials**

**Table S1.** *Categorical variable of the risk factor variables*

| **Socio-**  **demographic risk** | **Variable name** | **Original text** | **Categorical variable** |
| --- | --- | --- | --- |
|  | Age | “What is your age?” | *age* (20-44 years vs. 45-59 years vs. ≥60 years) |
|  | Gender | “What is your gender?” | *gender* (male vs. female) |
|  | Body mass index (BMI) | BMI was computed from dry weight in kilograms divided by height in meters squared (kg/m^2^) 1=normal weight (18.5≤ BMI < 23.9 kg/m^2^); 2=underweight (BMI < 18.5kg/m^2^); 3=overweight (24.0 ≤BMI <27.9 kg/m^2^); 4= obese(BMI ≥ 28.0 kg/m^2^). | *BMI* (normal weight (18.5≤ BMI < 23.9 kg/m^2^) vs. underweight (BMI < 18.5kg/m^2^) vs. overweight (24.0 ≤BMI <27.9 kg/m^2^) vs. obese (BMI ≥ 28.0 kg/m^2^) |
|  | Education | “What is your highest education?”; 1 = ≤ 6 years of schooling, 2 = 7-9 years of schooling, 3 = 10-13 years of schooling, 4 = Completed vocational studies, 5 = Completed studies, 6 = Doctorate | *Education* (less than high school (≤6 years of schooling) vs. high school (7-9 years of schooling) vs. higher school (≥10 years of schooling)) |
|  | Economic status | “What is your current total monthly household income?"1=<5,000 RMB/mo, 2=5,000-8,000 RMB/mo, 3=8,000-15,000 RMB/mo, 4= >15,000 RMB/mo | *Economic status* (<5,000 RMB/mo, vs. 5,000-8,000 RMB/mo, vs. 8,000-15,000 RMB/mo, vs. >15,000 RMB/mo) |
|  | Marital status | “Do you have a spouse now?”; 1 = Yes, I am married, 2 = Single, 3 = Unmarried, 4 = Divorced | *Widowed* (widowed vs. married, single, divorced) |
|  | Being childless | Do you have any children?  1 = Yes, 2 = No | *Being childless* (vs. having children) |
|  | Living situation | “Do you live together with other people?”; 0 = No, 1 = Yes | *living alone* (vs. not living alone) |
| **Health-related risk** | Smoking | “Do you have a habit of smoking?”; 1 =Current, 2 = Never, 3 = Former | *smoking status* (current vs. never vs. former) |
|  | Drinking | “Do you have a habit of drinking?”; 1 = Drinking daily, 2 = Drinking occasionally, 3 = Drinking in the past, 4=  Never drinking | *drinking status* (never or former vs. <7 d/wk vs. daily) |
|  | Fall History | “Have you experienced a fall in the past year?”; 0 = No, 1 = Yes | *Fall history* (vs. no) |
|  | Malnutrition | Malnutrition Inflammation Score (MIS) was used to evaluated the nutritional status, 0 = MIS < 6 points, 1 = MIS ≥ 6 points | *Malnutrition* (vs. no) |
|  | Charlson Comorbidity Index (CCI) | CCI was conducted to evaluate the chronic disease condition. CCI forms a composite score based on the presence of 19 comorbidities and is used to describe multiple comorbidities. 0=CCI ≤ 4, 1=CCI > 4). | *CCI* (0-4 vs > 4) |

| **Table S2.** *Latent class analysis - sensitivity analysis with all cases (N = 1136)* | | | | | | | | |
| --- | --- | --- | --- | --- | --- | --- | --- | --- |
|  | LL | AIC | BIC | aBIC | Entropy | LMR | BLRT | Class size and assignment probability |
| 1 class | -5881.225 | 11780.450 | 11825.767 | 11797.180 | - | - | - | 1090(100%) |
| 2 class | -4990.709 | 10019.418 | 10115.088 | 10054.739 | 0.819 | <0.001 | <0.001 | 422(37.15%)/714(62.85%) |
| **3 class** | **-4861.058** | **9780.117** | **9926.139** | **9834.027** | **0.779** | **<0.001** | **<0.001** | **144(12.68%)/419(36.88%)/573(50.44%)** |
| 4 class | -4830.543 | 9739.085 | 9935.461 | 9811.585 | 0.769 | <0.001 | 0.523 | 419(36.88%)/158(13.91%)/48(4.23%)/511(44.98%) |
| 5 class | -4801.029 | 9700.029 | 9946.757 | 9791.118 | 0.713 | 0.082 | 0.959 | 104(9.16%)/196(17.25%)/55(4.84%)/321(28.26%)/460(40.49%) |

Notes. LL, Log-likelihood; AIC, Akaike Information Criterion; BIC, Bayesian Information Criterion; aBIC, Adjusted Bayesian Information Criterion; LMR, Lo-Mendell-Rubin adjusted likelihood ratio；BLRT, bootstrap likelihood ratio test

**Table S3.** *Multiple group analyses to examine differences between the profiles in depressive symptoms using the chi-square test*

| **Chi-square tests** | *χ²* | *p* |
| --- | --- | --- |
| **PHQ-9** |  |  |
| Overall test | 432.13 | <0.001 |
| Class 1 vs. 2 | 148.68 | <0.001 |
| Class 1 vs. 3 | 394.32 | <0.001 |
| Class 2 vs. 3 | 98.40 | <0.001 |

*Notes.* PHQ-9: Patient Health Questionnaire 9. Class 1: *High depressive symptoms*. Class 2: *Moderate depressive symptoms.* Class 3: *Low depressive symptoms.*
